# Individualising Chronic Care Management by Analysing Patients’ Needs – A Mixed Method Approach

**DOI:** 10.5334/ijic.3067

**Published:** 2017-11-13

**Authors:** P. Timpel, C. Lang, J. Wens, JC. Contel, A. Gilis-Januszewska, K. Kemple, PE. Schwarz, on behalf of the MANAGE CARE Study Group

**Affiliations:** 1 Prevention and Care of Diabetes, Department of Medicine III, Faculty of Medicine Carl Gustav Carus, Technische Universität Dresden, Dresden, DE; 2 Department of General Practice, Department of Medicine III, Faculty of Medicine Carl Gustav Carus, Technische Universität Dresden, Dresden, DE; 3 Department of Primary and Interdisciplinary Care Antwerp, University of Antwerp, Antwerp, BE; 4 Chronic Care Program, Department of Health, Integrated Health and Social Care Plan, Generalitat de Catalunya, ES; 5 Department of Endocrinology, Jagiellonian University, Medical College, Kraków, PL; 6 Paul Langerhans Institut Dresden, German Center for Diabetes Research (DZD), Dresden, DE

**Keywords:** unmet patient needs, chronic care, type 2 diabetes mellitus, prevention and health promotion, integrated care

## Abstract

**Background:**

Modern health systems are increasingly faced with the challenge to provide effective, affordable and accessible health care for people with chronic conditions. As evidence on the specific unmet needs and their impact on health outcomes is limited, practical research is needed to tailor chronic care to individual needs of patients with diabetes. Qualitative approaches to describe professional and informal caregiving will support understanding the complexity of chronic care. Results are intended to provide practical recommendations to be used for systematic implementation of sustainable chronic care models.

**Method:**

A mixed method study was conducted. A standardised survey (n = 92) of experts in chronic care using mail responses to open-ended questions was conducted to analyse existing chronic care programs focusing on effective, problematic and missing components. An expert workshop (n = 22) of professionals and scientists of a European funded research project MANAGE CARE was used to define a limited number of unmet needs and priorities of elderly patients with type 2 diabetes mellitus and comorbidities. This list was validated and ranked using a multilingual online survey (n = 650). Participants of the online survey included patients, health care professionals and other stakeholders from 56 countries.

**Results:**

The survey indicated that current care models need to be improved in terms of financial support, case management and the consideration of social care. The expert workshop identified 150 patient needs which were summarised in 13 needs dimensions. The online survey of these pre-defined dimensions revealed that financial issues, education of both patients and professionals, availability of services as well as health promotion are the most important unmet needs for both patients and professionals.

**Conclusion:**

The study uncovered competing demands which are not limited to medical conditions. The findings emphasise that future care models need to focus stronger on individual patient needs and promote their active involvement in co-design and implementation. Future research is needed to develop new chronic care models providing evidence-based and practical implications for the regional care setting.

## Background

Today’s health systems are increasingly faced with the challenge to provide effective, affordable and accessible health care for people with chronic conditions. The growing number of people with chronic illnesses like diabetes is placing high burdens on health systems, society and individuals [1,2]. Diabetes prevalence in Europe is estimated to be 9.1% of the population aged 20–79. By 2040, more than one in ten European citizens will suffer from diabetes (10.1%) [3]. Diabetes care is costly for both individuals and health systems and requires an efficient management of expertise and resources. The involvement of patients, informal caregivers and professionals at a certain point in time is crucial to develop an individualised care plan [3,4,5]. It is essential to tailor this care plan according to the cultural background and local resources of the patient [6,7].

However, patient needs are diverse and complex. Patient-centred diabetes care and prevention involves lifestyle modifications, pharmacological interventions, as well as self-management education and support [8,9]. Self-management competences heavily depend on patients’ health literacy [10,11], physical abilities [12], cognitive impairments [13,14,15], socioeconomic status [16,17] and motivation [18], which indicates complex interrelations. Yet, most current treatment models and guidelines are disease and symptom focussed and do not seem to take these specific needs and priorities into account [19,20,21,22]. There are numerous definitions of patient needs following medical, sociological or political perspectives [23,24,25]. In the present study, a widely defined but patient-centred approach is used. Patient needs are seen as *“requirements of individuals to enable them to achieve, maintain or restore an acceptable level of social independence or quality of life”* [26].

A first scientific overview of the authors before conducting the present study revealed that systematic data on the strengths, weaknesses and limitations of existing chronic care programs is limited. Additionally, there is insufficient independent scientific literature investigating personal experiences, challenges and barriers in chronic care. Consequently, validated measures and instruments are mostly missing, although new instruments to assess patients’ “experience of care” have been recently developed [27]. A systematic review to investigate the effectiveness of chronic care models for the management of type 2 diabetes mellitus (T2DM) in Europe concludes that there is only limited evidence from European cluster randomized controlled trials [28].

To fill this gap this study explores, describes and ranks unmet needs in patients with T2DM and comorbidities regarding care and management of T2DM in different countries across Europe. The data will be integrated in an innovative and patient-centred model for optimising care and management of people with T2DM.

## Method

**Figure 1:**
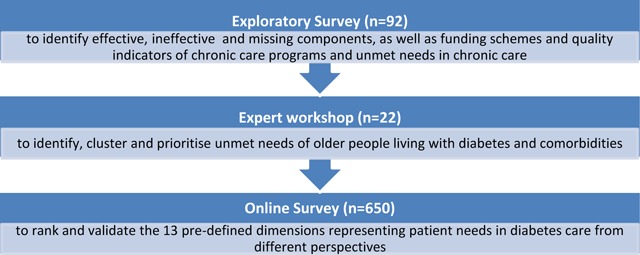
Multiple step approach to identify and rank unmet patient needs.

To identify and rank patient needs a *multiple step approach* was chosen (Figure 1). An **exploratory survey** was conducted from October to November 2014. The survey was generated by using findings of a desk research analysing international chronic care programs and results derived from a systematic review investigating the effectiveness of existing models [28]. Using a scientific multistep development process questions were generated and reviewed by a group of project partners of the EU-funded project MANAGE CARE. The final survey included eleven open-ended questions. Based on the scientific experience and the professional networks of the project partners the exploratory survey was distributed to experts and scientists in the field of chronic care management via e-mail. We received 92 completed questionnaires derived from 15 different countries (response rate: 36.4%). The open-ended questions included reflections on effective, problematic and missing components of established chronic care programs in the specific countries of the experts and scientists. Multiple answers were allowed. An empirical content analysis, data coding and indexing was applied to analyse the qualitative data and enabled the representation of cumulative frequencies.

An **expert workshop** in March 2015 included 22 experts of the MANAGE CARE project with a strong background in patient management, integrated chronic care, diabetes research and health policy. Using the overview derived from the findings of the exploratory survey, the workshop was used to identify unmet needs of elderly people living with T2DM and its comorbidities. Participants were asked about barriers, difficulties and challenges in the disease management system. The qualitative answers were summarised and discussed using a Delphi-like procedure. Finally, a prioritisation round was set up reflecting on the four perspectives health, patient, health care professionals (HCP), and health care service (HCS) to differentiate the impact of the needs dimensions. The “health perspective” was used as an umbrella term with a significant public health focus. The “patient perspective” was used to evaluate the influence of unmet needs on the individual patient, especially affecting activities of daily living, participation and quality of life. The prioritisation of components was done by every expert using “1” with the highest and “13” with the lowest priority resulting in calculated means. A comparison of means was applied to rank the dimensions. Prioritisation from lowest (red) to highest (green) priority was applied to the four perspectives.

An **online survey** was developed to validate the results of the expert workshop. This survey was carried out from April to August 2016 using a drag-and-drop method, in which the participants select an object by clicking on one of the thirteen needs dimensions and move it to the preferred position of the ranking. This simple and intuitive method was available in 11 languages. Patients, health care providers and other stakeholders (n = 650) involved in chronic care ranked the 13 pre-defined dimensions. A comparison of ranks, medians and means was applied to identify priorities and to compare the results of the expert workshop with the findings of the online survey. The Mann–Whitney U-test as the non-parametric alternative test to the independent sample t-test was used since ordinal data was analysed. Statistical analyses were performed using SPSS for Windows version 23.0 (SPSS, Chicago, IL). The statistical significance level was set at 5%.

## Results

### Exploratory Survey

The aim of the exploratory survey was to investigate effective, problematic and missing components in existing chronic care programs, funding and quality measurement of these programs. Ninety-two experts from 15 countries participated: Germany (33%), Belgium (8%), China (8%), and 4% each from Serbia, Israel, Hungary, Spain, Ireland, Lithuania, Albania, Georgia, France, Russia, Denmark and Italy. More than 40% of the participants were health professionals, followed by researchers (19%), patients (14%), program leaders (13%), patient and diabetes organisations (8%) and others (7%).

When asked for *effective components* (Table 1) of existing chronic care programs implemented in the specific countries of the respondents the most mentioned item was “Diagnosis and Treatment” (79.2%). Participants justified their answers by arguing that this component is irreplaceable regarding early detection through screening, early treatment of diabetes complications, new medication for diabetes treatment, and check-ups. This item is followed by “Education of patients and health care professionals” (75%). Almost two thirds (62.5%) stated that “Multi- and Interdisciplinarity” is an effective part in chronic care programs.

**Table 1:** Exploratory Survey – Effective components of chronic care programs.

Item	%

Diagnosis and Treatment	79.2%
Education of Patients/HCP	75.0%
Multi-/Interdisciplinarity	62.5%
Monitoring and Self-Control	58.3%
(Lab) Parameter Control	45.8%
Guideline Adherence	25.0%
Lifestyle Change	12.5%
Involving Public Health Institutions	12.5%
Diabetes Registry	8.3%
Pay-for-performance; Accreditation; Easy Access, Reasonable Price; DMP	4.2% (each)

“Inadequate health care” was rated as the most *problematic component* (Table 2). Participants summarised a lack of national guidelines, poor decision making processes, cost-ineffective health care, lack of alternative medication, long waiting periods, limited opportunities for individualised treatment, and inadequate tools for physicians under this category. Insufficient payment and financial coverage was ranked as second, followed by inadequate governmental structures with both more than 50%. The category funding refers to the summarised qualitative data, stating that funding was not part of the respective program, restricted lists for reimbursements limit freedom for treatment and prevention was not a priority in the budget. These findings suggest that system-related mechanisms beyond the influence of the health care provider are identified as most hampering components to deliver chronic care.

**Table 2:** Exploratory Survey — Problematic components of chronic care programs.

Item	%

Inadequate Health Care	76.2%
Insufficient Payment/Funding	57.1%
Incompetence of governmental structures/HCP	52.4%
Unclear or Lack of Data	47.6%
Insufficient Collaboration/Networking	38.1%
Insufficient Adherence/Compliance of Patients	28.6%
Increased Bureaucracy	19.0%
Patient-related barriers	9.5%

**Figure 2:**
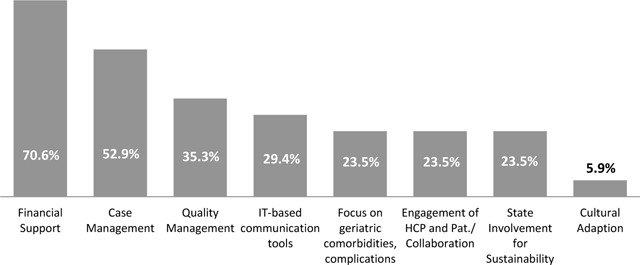
Exploratory Survey – Missing components of chronic care programs (n = 93).

The participating experts stated that “Financial support” (no tangible incentives, scarcity of funding, and no refund of diagnostics and lab controls; 70.6%) followed by “Case Management” (ensuring integrated and around-the-clock care, individual treatment, and prevention; 52.9%) and “Quality Management” (evaluation and standardised measures, quality of life-measures, assessments on individual levels and process flows; 35.3%), seem to be missing in established care programs (Figure 2). In addition, almost 30% argued that “IT (information technology)-based communication tools” such as telemedicine are lacking.

**Figure 3:**
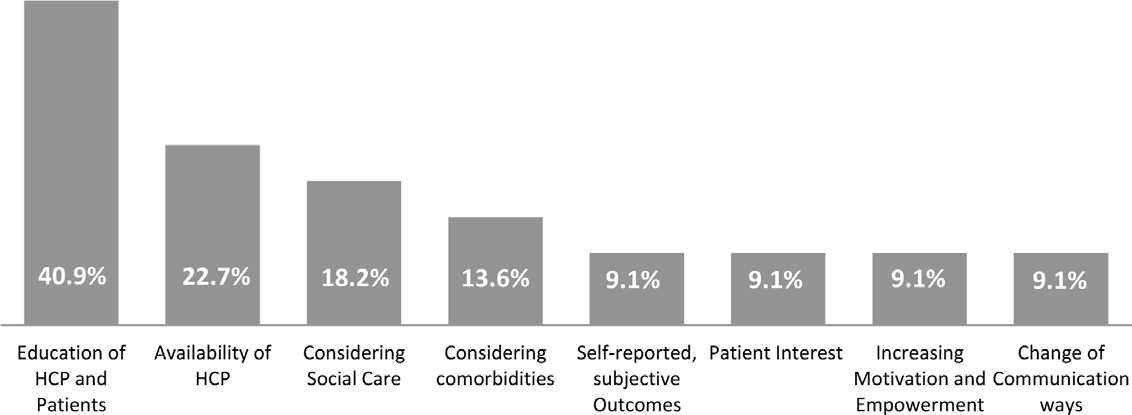
Exploratory Survey – Unmet needs and priorities (n = 93).

Education of health care professionals and patients (40.9%) and availability of health care professionals (22.7%) were revealed as patients’ needs and priorities which remain unmet in established chronic care programs (Figure 3).

### Expert workshop

**Figure 4:**
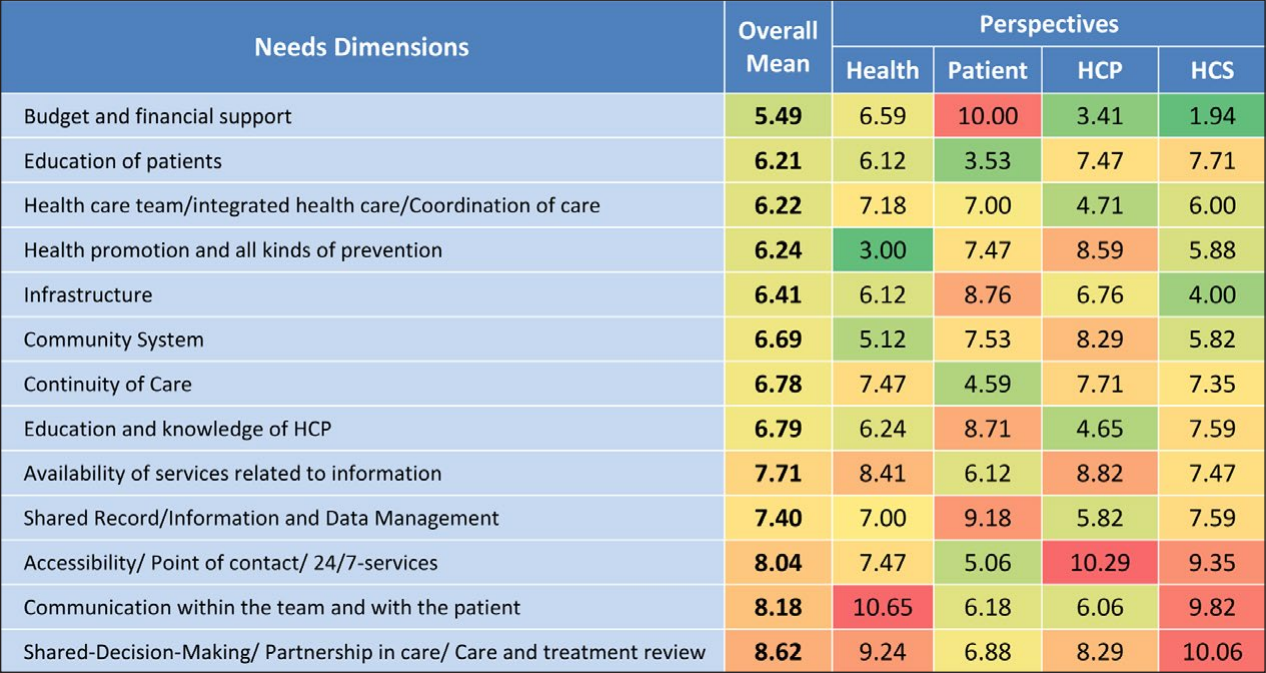
Expert workshop – Ranked exercise results showing priorities of need dimensions. The 13 identified key dimensions reflect patients’ needs in chronic care. Experts (n = 22) ranked the items from 1 (highest priority, green) to 13 (lowest priority, red). Numbers indicate means of the dimensions according the relevant perspective.

The expert workshop resulted in a list of 150 unmet needs, which were discussed, summarised and classified into 13 dimensions (Figure 4). The results indicate that according to the experts’ views for the same components, priorities differ considerably depending on the perspective. “Budget and financial support” was rated as the need dimension with the highest priority for HCPs (3.41) and health care services (1.94), but with the lowest for patients (10.0). Highest overall mean value was rated for “Shared decision making” (8.62).

From the **health perspective**, the lowest priority was given to the component “communication within the team and with the patient” (10.65). On the other hand, community based aspects of “health promotion and all kinds of prevention” was ranked at the highest priority.

Looking from the **patient perspective**, “Education of patients” was given the highest priority (lowest mean: 3.53). In contrast to the system and professionals’ perspectives, “budget and financial support” scored the lowest priority (10.0) as an unmet need.

Highest priority from the **health care professional’s perspective** was given to “Budget and financial support” (3.41). Seen from the **health care services’ perspective**, the highest priority within the whole survey was given to costs (HCS mean 1,94) followed by the importance of available “Infrastructure” for chronic care delivery (4.0). Surprisingly, the lowest priority was given from the services’ perspective to “Shared decision making/Partnership in care/Care and treatment review” (10.06) and “Communication within the team and with the patient” (9.82).

### Online survey

The online survey included 650 participants derived from 56 countries after data cleaning. Data series of 19 participants were excluded due to redundant or incorrect data or noted test files. Participants included 341 HCPs (52.5%), 277 patients (42.6%) and 32 stakeholders of health service units (4.9%).

Participants from Portugal and Poland accounted for more than half (57.5%) of the whole study population (Table 3), because the survey invitation was spread in the Portuguese Diabetes Association (Associação Protectora dos Diabéticos de Portugal – APDP) and disseminated among more than 3.000 physicians via the general practitioners’ organisation “Kolegium Lekarzy Rodzinnych” in Poland.

**Table 3:** Online Survey — Frequency table of participating countries (n = 650).

Country	Frequency	Percent

Portugal	212	32.6
Poland	162	24.9
Greece	79	12.2
Germany	38	5.8
Spain	36	5.5
Serbia	15	2.3
Great Britain	10	1.5
LAT	9	1.4
Austria	8	1.2
Belgium	6	0.9
Italy	6	0.9
Others (<6)	69	10.6

#### Statistical data analysis – Mann-Whitney-U-Test

**Figure 5:**
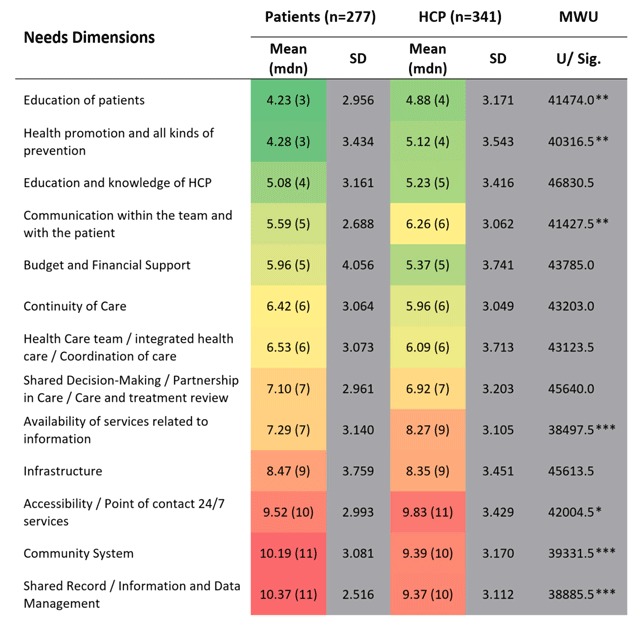
Online Survey – Ranking of means for pre-defined needs dimensions comparing patients’ and HCP’s views. *p < 0.05, **p < 0.01, ***p < 0.001. Prioritisation of needs using “1” as the highest (green) and “13” as the lowest (red) priority resulting in calculated means.

Mann-Whitney-U-Test was performed to test whether the two samples have the same shape, i.e. by comparing the medians between them. Participants representing health care units were excluded from the group analysis due to low participant rates. Figure 5 shows the ranked means (M), medians (mdn) and standard deviations (SD) for patients (n = 277) compared to HCPs (n = 341). “U” reflects the differences between the rank totals.

Two key messages can be drawn from Figure 5: (1) The mean and median for the ranked patient needs enable to draw an overall ranking of needs dimensions with high and low priorities, while (2) the MWU-test allows accounting for group-specific differences between patients and HCPs in the ranking of one needs dimension.

“Education of patients” was rated in total as the most important patient need for both patients (*M* = 4.23, *SD* = 2.956) and HCPs (*M* = 4.88, *SD* = 3.171), followed by “Health promotion and all kinds of prevention” and “Education and knowledge of HCP”. Lowest priority was given to “Shared record/Information and Data Management” (*M* = 10.37, *SD* = 2.516) by patients whereas HCPs ranked “Accessibility/Point of contact 24/7 services” with the lowest importance (*M* = 9.83, *SD* = 3.429).

The overall ranking of dimensions stays mostly the same even though ranks differ between patients and professionals for some dimensions. SD are highest for “Budget and financial support” and “Infrastructure” for patients indicating inhomogeneous answers. The comparison of the items with the highest and the lowest mean value for patients (6.14) and HCPs (4.49) shows a smaller range for HCPs rankings.

#### Output and interpretation

Figure 5 shows statistically significant differences of medians between patients and health care professionals for e.g. “Education of patients” (*p* < 0.01), “Health promotion and all kinds of prevention” (*p* < 0.01) and “Communication with the team and with the patient” (*p* < 0.01). All three needs dimensions are significantly more important to patients.

Regarding the midfield of both rankings, aspects of the care process like continuity, coordination and treatment review are present. “Availability of services related to information” shows the only significant median difference for patients (*Mdn* = 7) and health professionals (*Mdn* = 9, *p* < 0.001).

System-related aspects of unmet needs are listed at the bottom of the table, indicating lower importance for both groups in contrast to aspects reflecting communication and education as well as the care process. Nevertheless, financial issues are very important to both patients and HCPs (*Mdn* = 5). Differences in the lowest third of the two rankings are significant for “Community system” (*p* < 0.001) and “Shared record/information and data management” (*p* < 0.001). Both needs dimensions are significantly more important to Health professionals (*Mdn* = 10) compared to patients (*Mdn* = 11). The accessibility of services – as the least important unmet need from the professionals’ perspective (*Mdn* = 11) – has a significantly higher priority for patients (*Mdn* = 10, *p* < 0.05).

Due to the high amount of participants from Poland (24.9%) and Portugal (32.6%) two additional MWU-Tests were performed. The aim was to test for differences between patients from Portugal compared to other countries as well as for professionals from Poland compared to other countries. The analyses are illustrated in Tables 4 and 5.

**Table 4:** Online Survey – Country-specific ranking of means: HCPs Poland vs. other countries.

Needs Dimensions	Poland (n = 139)		Other countries (n = 202)		MWU
	
	Mean (*mdn*)	*SD*	Mean (*mdn*)	*SD*	U/Sig.

Continuity of Care	5.50(5)	2.824	6.27(6)	3.164	12180.5*
Health Care team/integrated health care/Coordination of care	6.77(6)	3.986	5.62(5)	3.433	11709.5**
Budget and Financial Support	4.66(4)	3.329	5.85(5)	3.935	11707.5**
Accessibility/Point of contact 24/7 services	10.53(12)	3.326	9.31(10)	3.297	10282.0***

*p < 0.05, **p < 0.01, ***p < 0.001.

**Table 5:** Online Survey – Country-specific ranking of means: patients Portugal vs. other countries.

Needs Dimensions	Portugal (n = 203)		Other countries (n = 74)		MWU
	
	Mean (*mdn*)	*SD*	Mean (*mdn*)	*SD*	U/Sig.

Health promotion and all kinds of prevention	3.82(3)	3.244	5.55(6)	3.634	5306.0***
Accessibility/Point of contact 24/7 services	9.91(11)	2.732	8.47(9)	3.421	5667.5**

*p < 0.05, **p < 0.01, ***p < 0.001.

The country-specific analyses underline that priorities for some needs dimensions differ between countries. HCPs from Poland (n = 139) tend to have different priorities of patients’ needs regarding financial aspects and the care process. Continuity of care (*Mdn* = 5/6, *p* < 0.05) and financial issues (*Mdn* = 4/5, *p* < 0.01) are in contrast more important to the professionals from Poland. In terms of accessibility of services the lower ranking of Polish HCPs (*Mdn* = 12) was highly significant when compared to other countries (*Mdn* = 10, *p* < 0.001).

Table 5 illustrates similar rankings except for two significant median differences between patients from Portugal (n = 203) and those coming from the other 55 countries (n = 74). Preventive issues are significantly more important for Portuguese patients (*Mdn* = 3) when compared to patients with other national backgrounds (*Mdn* = 5, *p* < 0.001). On the other hand, adequate accessibility of services has a lower priority for patients from Portugal (*Mdn* = 11) compared to others (*Mdn* = 9, *p* < 0.01).

## Discussion

### Reducing complexity to take action

Our results show that unmet needs are seen as a complex concept relying on multiple (often competing) demands, expectations and interpretations. This is also supported by the findings of three analytical studies that support the concept that chronic care has to direct more attention on individual needs of patients and individuals at risk [29,30]. Our findings also suggest that chronic patients are faced with unmet needs which are not limited to medical conditions [31]. Many participants of the presented survey are convinced that social care (18%) and comorbidities (14%) are inadequately considered in the current chronic care programs. The incorporation of social services and informal social support, especially for people with complex health and social care needs, is also strongly recommended by many experts [32,33].

The multiple-step approach shows that the players involved in chronic care, especially patients and healthcare professionals, share a similar understanding to tailor interventions to individual needs. However, analyses proved that priorities of various patient needs as well as the overall range differ between patients and HCPs. One interpretation for the higher range of patients’ rankings could be that professionals tend to see the “bigger picture” of chronic care, including its complexity and factors determining the own care agenda (e.g. financing and care delivery). This could have led to a more balanced ranking of professionals. These findings are supported by Drennan et al., as the “patient perspective” might be more defined by aspects reflecting patients’ autonomy and the increase of their own leeway for action [30].

### Patient education, communication and prevention in chronic care

Patient education and involvement is of key importance for both groups. This is also supported by Sinclair et al. (2010), highlighting that diabetes education is indispensable for both patients and professionals [34]. Data derived from the expert workshop and the online survey suggest that needs related to education and communication are more important to patients.

Recent findings indicate that the combination of prevention and chronic care supports access and participation, especially for high-risk populations and vulnerable target groups [35]. This is especially important for those patients having little or no awareness of what to do when they are experiencing hypoglycaemia. Additionally, findings indicate that the same group of patients rarely or never talk to their GP/specialist about their hypoglycaemia [36,37], which underlines the importance of communicative competences identified in our study.

The expert workshop seems to have underrated the value of “Health promotion and all kinds of prevention”. From the “health perspective” the lowest priority was given to the component “Communication within the team and with the patient”, perhaps because in the early phase of chronicity, e.g. in case of newly diagnosed patients, no “team” around a “patient” is set up yet, where in the same understanding “Shared decision making/Partnership in care/Care and treatment review” was also ranked with low priority.

### Financial and structural barriers

Other studies reported that inadequate financial coverage, limited availability of services and conflicting structural or legal constraints may result in not fully covered costs of implementation at the practice [2,38]. Our findings concurred with the previous studies, as system-related aspects, especially cost issues, showed a high priority within both user groups. Although financial matters for patients were underrated by experts, more than 70% of the experts in the exploratory survey responded that financial support is the most important missing component in chronic care programs.

Next to financial barriers, the availability of services seems to hamper adequate and accessible care. The present study shows that availability of services is more important for patients (*Mdn* = 7) compared to professionals (*Mdn* = 9, *p* < 0.001). Possibly, patients fear to have an urgent need for care when there is no service available or it is impossible to be reached. The low rank of accessibility in both groups as a result of the online survey is surprising though. A possible explanation could be that 24/7 access is more important to patients with multimorbid and complex conditions than well-managed diabetics. Access and adherence to diabetes medication can be limited by costs, leading to a higher risk of inadequate care or underuse of medication [39,40,41]. Other studies support that geographical distance and shortage of services may increase patients’ vulnerability [42], or the risk of receiving inadequate health care [43,44].

### Sharing data and information

As almost half of the participants in the exploratory survey (47.6%) stated that unclear information or lack of data was hampering success of established chronic care programs, it is surprising that sharing of data was ranked with a very low priority by the professionals in the online survey. It was also expected that patients would rank it with a higher importance as they empirically have an interest that information is shared to avoid doublings of assessments and prevent loss of information between providers and sectors.

### Community support and basic unmet needs

Evidence shows the high potential of supportive communities [45] but also indicates the negative impact of diabetes on family members of people with diabetes [46]. Setting up robust care and support in the private household by involving informal care givers and the local community can be complex. Consequently, elderly diabetic patients who receive home health care and post-discharge services have a risk for specific unmet needs related to social work services, home health aid, homemaker services, and need for medical equipment [47]. A systematic review of Barnard et al., summarises unmet basic needs like medication, housing, transportation and telecommunication which are of clinical importance and affecting diabetes outcomes [48]. The role of communities in chronic care was rated with a high priority during the expert workshop. However, patients and professionals ranked the community system in the online survey surprisingly with a very low priority. Additionally, the community system including professional and informal support was given a higher priority by professionals. One explanation could be that HCPs have a more holistic perspective on health care delivery than patients. It is likely that patients tend to evaluate needs having a direct impact on their daily care, making the potential of communities seem less relevant. A second explanation could be that different types of patients with diabetes, either related to elderly or complex conditions, affect the ranking. In fact, evidence shows that linking community resources with health care delivery system redesign and self-management support can help patients to improve diabetes control [45]. However, not considering the living environment underestimates potential barriers that especially rural populations are faced with [49]. As an example, chronic patients in rural areas are often faced with a double jeopardy due to an increase of demand for care, ageing and declining of communities [50].

### Country-specific approaches are needed

Professionals from Poland stated significantly higher priorities for unmet needs relating to budget issues, care coordination and continuity, but lower importance for accessibility of services. Other sources report that waiting times do restrict access to healthcare being the second highest in the EU. Secondly, unmet medical needs for reasons of cost are also above the EU average [51]. Another explanation for these differences could be inequalities in health associated with socioeconomic status [52].

Patients from Portugal ranked prevention and health promotion on a very high level, whereas accessibility of services was ranked with a lower priority compared to other countries. A recent report by the International Diabetes Federation (IDF) supports these findings, saying that access to education and information was generally good in Portugal compared to other European countries [53]. A current EU report states that experiences of patients in primary care differ tremendously between countries. The age-standardised rates for easy-to-understand explanations by the doctor, opportunity to ask questions and involving the patients in terms of decisions about care and treatment were seen more positive by patients from Portugal compared to those from Poland [54]. The proportion of people reporting unmet needs due to financial reasons among the low-income tripled in Portugal between 2008 and 2014, raising concerns that they may result in poorer health status and increased health inequalities [54].

Country-specific results from the online survey showed that priorities for some dimensions differ for the origins of the participants. However, the country-specific analysis has to take into account the context and setting, including country, region, available services/networks and existing resources, in which the care models are applied.

### Integrating patient needs in sustainable case and care management strategies

Comorbidities can have profound effects on both the professional care and case management and the individual self-management of patients leading to competing demands [55,56,57]. According to the American Diabetes Association (ADA) treatment decisions should be timely, evidence-based, as well as tailored to individual patient preferences, prognoses, and comorbidities [7]. However, it is likely that patient’s priorities differ between patients where diabetes is an index disease and those cases where multimorbid patients suffer from complex health and social needs. Participants of the survey stated a strong need for case and quality management. These findings are supported by research studies, underlining the need for personalised, not for personal care [58]. In this respect, qualitative findings suggest that patients with diabetes are exposed to a variety of fears and needs, related to the diagnosis, treatment, expected impacts, prognosis and the daily management of the disease [59]. Supporting this, the study 3DfD (3 Dimensions of care for Diabetes), representing an award-winning model of integrated care, integrates social support with diabetes and mental health care [60]. Diabetes management for patients with poor glycaemic control can be improved by supporting collaborative care planning and dynamic adjustment of care objectives [61,62]. This is also true for behaviour change [63]. Our study shows that shared decision making and communication with the patient and team are highly desired by all user groups. Evidence from other studies supports that the effectiveness of interventions for patients with chronic diseases is improved when collaborative care models are applied which are based in the community or supported by proactive case management [64].

### Strengths and Limitations of the Study

The present study uses a mixed-method approach to analyse unmet needs of patients in chronic care with special focus on T2DM resulting in a practical overview. The multiple methods used support the power of the findings. The two exploratory and descriptive survey designs are based on both systematic findings [65] as well as strong practical and international expert opinions. The methods applied were used to investigate and rank the identified needs from four perspectives: health, patient, provider and health system. Using multiple perspectives on a European level helps tremendously to generate strong but at the same time not regionally limited evidence.

The study is not suitable to provide qualitative insights into detailed care regimes and their impact on process indicators or multiple health outcomes. The applied overview of patient needs is not exhaustive and needs further investigation as well as systematic approaches.

The study design, especially of the online survey, was set up in a user-friendly way. Different variables like age, cultural background, socioeconomic status, comorbidities, years since diabetes diagnose (for patients) and proportions of patients with diabetes (for professionals) were not considered. Due to the minimalistic design – online ranking of 13 dimensions – there is a potential selection bias that patients with digital competences were more likely to participate and some dimensions might be unknown to some participants. This could be the case for those who are not familiar with these terms like patients and providers not actively involved in the care process. Additionally, the applied method forced participants to rank needs dimensions although it is likely that differences between some items might not have been intended by the participants. The number of participants included in the explanatory and the online survey is not suitable to develop evidence-based recommendations. However, the study design and the participant numbers provide a first overview about the variety of patient needs and their individual priorities.

## Conclusion and areas for further research

The presented results underline that patient needs are individual, not limited to medical domains and should become a cornerstone for the development of chronic care management models. Financial issues, education of both patients and HCPs, access and availability of services as well as health promotion are the most important patient needs seen by patients and professionals. However, they will have to be accompanied by several innovative actions on individual and environmental level to slow down the progression of T2DM, in addition to approaches focusing on education and counselling [66].

Future research should also investigate processes and potential for innovation on system-level and incorporate the findings in new sustainable models for individualised disease management for patients with diabetes and pre-diabetes [67]. These new models should empower HCPs in a practical way to translate the identified needs into patient-centred care plans and to evaluate outcomes and perceptions using measures that are not only medically driven.

The multi-method research applied – in which different point-of-views were placed against each other – explored new priorities for further research. The study supports that identifying and prioritising new areas of research together with patients and citizens is a promising empirical approach.
